# Letter from Gardner, Ill.

**Published:** 1883-09

**Authors:** C. M. Easton

**Affiliations:** Gardner, Illinois


### ARTICLE XIII.

GARDNER, ILLINOIS, July 18, 1883.

EDITORS CHICAGO MEDICAL JOURNAL AND EXAMINER.

*My Dear Sirs:*—During the early part of last November a man came rushing into my office, nearly out of breath, telling me, "Come to my house as quick as possible, my boy is choking to death!" The place named was only three or four blocks away, and I lost no time in getting there; but death was there in advance. The mother and neighbors told me, "the child did not breathe after the father started." The boy was three years old, well developed, and was playing on the floor with other children, when, the play not going to suit, he began to cry, which brought on a paroxysm of coughing, then choking with results as stated.

The little fellow had had whooping-cough for some time, but was getting better. The history pointed to occlusion of the air passage as the cause of the sudden death, but as the child had nothing in its mouth at the time, I could not account for it, and was not a little exercised over the matter. The next day, having gained the consent of the parents, in the presence of the local fraternity, we made an examination, post-mortem, and found a chunk of fat pork in the trachea, immediately above the bifurcation. The mass completely filled the tube for the space of an inch and a half, and appeared as though it had been packed in there with considerable force.

The child died at five o'clock P. M. and had eaten nothing since dinner, about noon. The meat must have been swallowed at that time, and remained in the stomach until, during the fit of coughing, it was forced up into the pharynx. The sucking inspiration, peculiar to pertussis, drew it down into the trachea, causing sudden death.

We occasionally hear of some one being "choked to death;" sometimes on meat, but after being safely landed in the stomach and remaining there for five hours, we would hardly look for it in the windpipe. C. M. EASTON, M.D.

---

ARTICLE —.

QUINCY, ILLS., July 9, 1883.

DANIEL R. BROWER, M.D.

*Dear Doctor:*—Nearly a year ago I promised to write you an accouut of the results of the use of an anæsthetic mixture, used in a little operation for an exostosis upon the last phalanx of the great toe, where you kindly assisted me, you desiring to publish the account in the CHICAGO MEDICAL JOURNAL AND EXAMINER.

I should have written to you sooner, but wanted to report a sufficient number of administrations to give a reasonable idea of what might be expected from the use of the mixture.

I have now given it, or had it given fifty-seven times, with untoward results in but two cases, and in them every thing came out all right. Dr. C. B. Ellis, who has given it for me oftener than any one else, will explain why and how these symptoms appeared in his appended note.

Having lost a patient from the effects of chloroform several years since, I gave that up as an anæsthetic. Bromide of ethyl caused such intense redness of the face and such raving delirium that I gave that up Ether was followed so often by vomiting, and the time for the full recovery of consciousness was so long that I was not satisfied with it. To obviate these defects, I believed a mixture might be made, that would unite the good qualities of chloroform with the safety of ether. I first made a mixture of chloroform one part, bromide ethyl one part, and alcohol four parts. This, I hoped, would act well, since chloroform made the patients taking it very pale, and the bromide of ethyl made them very red. The alcohol was added as a diluent. In practice I found patients went under the influence in from one to four minutes, and came out in equally as short a time, but there was the deep flush and delirium, as when the bromide of ethyl alone was used.

The quantity of chloroform was increased until the mixture was: Bromide ethyl one part, chloroform three parts, alcohol four parts. When this mixture is administered the face retains its natural hue, the respirations and pulse are but slightly accelerated. There was but two of the fifty-seven patients who vomited; one of them, a very hystericy woman, very subject to vomiting, and the other had just eaten a hearty meal. I have given it nearly every time in a Dr. J. C. Hutchinson's (of Brooklyn) inhaler. Most of the patients did not require over two drachms to put them fully under its influence. Two ounces was as much as used in any operation, and that lasted for over an hour, and was an abdominal section for the removal of an extra-uterine fœtus. The operations have been for amputations, tracheotomy, abdominal section, crushing a stone in the bladder, enucleation of the eye, castration and minor operations. The cases have been selected, not so much on account of the anæsthetic, but on account of the person that was to administer the anæsthetic. When I was afraid that the administrator would take more interest in the operation than the patient, I have kindly permitted him to use ether in preference to the mixture, which was on its trial. Hoping that others may test it and find it as efficient and safe as I have, I remain

Very truly yours, WILLIAM A. BYRD.

---

